# Stakeholder engagement to develop a directory of COVID-19 related mental health services in Vietnam: reflections on a participatory approach

**DOI:** 10.12688/wellcomeopenres.20491.1

**Published:** 2024-02-19

**Authors:** Jessica Ngoc Thai, William Le Craig, Jaom Fisher, Mary Chambers

**Affiliations:** 1 College of Education, Psychology and Social Work, Flinders University, Adelaide, South Australia, Australia; 2 Imperial College London, London, England, UK; 3 Oxford University Clinical Research Unit, Ho Chi Minh City, Vietnam; 4 Nuffield Department of Medicine: Tropical Medicine and Global Health, University of Oxford, Oxford, England, UK

**Keywords:** Public engagement, stakeholder engagement, mental health, mental health support, mental health services, Vietnam, COVID-19

## Abstract

The COVID-19 pandemic led to a rise of mental health issues amongst Vietnamese communities in Vietnam and the diaspora. However, there were few resources and no directory of services available for people seeking mental health support in Vietnam. In response to this need, we initiated an engagement project to improve Vietnamese communities' access to mental health support. This project aimed to involve stakeholders in the development of a directory of resources in order to ensure that it met local needs. The phases of development included: (1) reviewing desk research findings; (2) reviewing the list of mental health support services that we could find; (3) verifying the first draft of the directory; (4) helping disseminate the directory; and (5) updating the directory. In February 2022 the first edition of the mental health directory for Vietnamese and foreigners living in Vietnam was published.

In this paper we describe the iterative approach taken to developing a resource that would have maximum utility for the target communities. We describe the process of partnering with people with lived experience, community members and expert stakeholders in this process, and reflect on how this strengthened the outcomes in terms of the relevance of the output, the research uptake and the access for the wider community. We believe that it is important to publish examples of community engagement projects in order to demonstrate good practise and promote increased involvement of communities in research.

## Plain Language Summary

The COVID-19 pandemic led to a rise of mental health issues amongst Vietnamese communities in Vietnam and those living overseas. However, there are few resources and no directory of services available for people seeking mental health support in Vietnam. In response to this need, the public engagement team at the Oxford University Clinical Research Unit in Ho Chi Minh city, Vietnam, worked with local experts and community groups to create a directory of resources available to support mental health. In February 2022, the first edition of the mental health directory for Vietnamese and foreigners living in Vietnam was published. In this paper, we describe the steps we took to developing the resource. We describe the process of partnering with people with lived experience, community members and expert stakeholders in this process, and reflect how their involvement helped create a more relevant resource.

## Introduction

On March 11, 2020, the World Health Organization (WHO) officially declared COVID-19, an infectious respiratory disease caused by the SARS-CoV-2 novel coronavirus, as a pandemic ( WHO, 2020a; WHO, 2020b). Since the first reported case (January 2020), until the time this project began (August 2021), there were over 4.3 million deaths worldwide. In Vietnam during this timeframe, there were over 3750 deaths and 224,800 COVID-19 cases reported ( JHU, 2021). Vietnam had four main waves of COVID-19 transmission. The fourth COVID-19 wave, commencing in late April 2021, led to a complete lockdown under the Government's Directive No. 16, imposed first in southern areas then to other regions ( Giang, 2021; Quang *et al.,* 2021). Continuing it’s Zero COVID policy, Vietnam’s government asked for closure of non-essential businesses and strict travel restrictions, and residents were prohibited from leaving their homes. In many places food supplies became scarce after 3 months of lockdown. Schools were also shut down for over a year, with online learning beginning in February 2021 and primary schools not reopening until after February 2022 ( ABC News, 2021; Lea, 2021; Manh, 2022; Phan, 2021).

Globally, adverse mental health outcomes due to the COVID-19 pandemic place multiple impacts on people’s lives at individual, family and community levels ( AIHW, 2020; Usher *et al.,* 2020). Vietnamese communities were no exception and experienced a range of adverse impacts on mental health due to the severe social distancing measures and the nationwide lockdowns. According to a survey regarding psychological impacts of COVID-19 during the first nationwide lockdown in Vietnam conducted in April 2020, 35.9% of the Vietnamese general population has suffered mental distress related to COVID-19 ( Duong *et al.,* 2020; Nguyen & Le, 2021). Prevalent mental health problems among Vietnamese communities involved anxiety, depression, emotional distress and suicidal ideation ( Chakraborty & Samuels, 2021; Duong *et al.,* 2020; ISDS and Rosa Luxemburg Stiftung SEA-Hanoi Office, 2020; Le *et al.,* 2020; Nguyen *et al.,* 2021; UN Vietnam, 2020).

Overseas Vietnamese communities may have the added mental burdens rooted from experiences of COVID-19 racial discrimination. Stigmatization was pervasive within Asian American groups, including Vietnamese – Americans and Vietnamese nationals, resulting from blame for the origin of the COVID-19 pandemic being in Asia ( Li & Nicholson, 2021; Om, 2020; Woo & Jun, 2021). Overseas Vietnamese populations, especially overseas Vietnamese students, also suffered COVID-19 mental distress due to Vietnam’s border closure. Travel restrictions during the pandemic constrained Vietnamese nationals from flying home and concurrently increased experiences of isolation and homesickness. It was reported that levels of anxiety, depression and pessimism were high amongst overseas Vietnamese communities ( Nguyen, 2021a; Nguyen, 2021b; Pham & Shi, 2020). In response to this need, a project to improve access to mental health support for Vietnamese communities in and outside of Vietnam was initiated. 

The involvement of stakeholders and communities in development and research initiatives is recognised to be an important step to ensure relevance and acceptance of the process and outcomes ( Boaz *et al.,* 2018; Chambers, 1992; Lenette, 2022). Trust is a key component of effective community/project partnerships ( Jagosh *et al.,* 2015; Vincent *et al.,* 2022) and collaborating with people and groups who already have relationships can be beneficial as it utilizes an existing trust ( Murphy *et al.,* 2021). This letter describes the participatory process of documenting mental health related services in Vietnam, and in particular reflects on the challenges and benefits of involving stakeholders throughout the process. 

### Project rationale

According to a UNICEF study in 2018, there was little knowledge of available mental health services in Vietnam. UNICEF (2018) found that respondents of their study had no information of where to seek professional mental health examination or support. According to our research in August 2021, there were no resources documenting Vietnamese service providers in the field of mental health. Thus, in the light of the increasing mental health problems related to the COVID-19 pandemic amongst Vietnamese communities, the Public and Community Engagement (PCE) Department of the Oxford University Clinical Research Unit – Vietnam (OUCRU) undertook to develop a directory of COVID-19 related mental health services in Vietnam. The project had 3 goals:

- Goal 1: To provide a broad overview of the current COVID-19 mental health problems, identifying key vulnerable groups, and identifying the gap in COVID-19 mental health services for Vietnamese communities;
- Goal 2: To create resources that summarise COVID-19 mental health stakeholders and services available for the Vietnamese communities and the expatriate community living in Vietnam;
- Goal 3: To support increased networking among Vietnamese mental health stakeholders.

Stakeholder engagement can play a crucial role in closing the gap between health research and practical implementation, and can help shape a project in line with the needs of service users ( Akwanalo *et al.,* 2019; Boaz *et al.,* 2018; Patil *et al.,* 2021). In order to compile a directory which can efficiently aid Vietnamese people to access mental health services and other health related support, multiple stakeholders were invited to be involved in all stages of the project.

***Literature review to identify Vietnam’s vulnerable groups and their COVID-19 mental health needs***

From August 2 to August 31, 2021 (stage 1 and stage 2), we undertook a literature review of the COVID-19 related mental impacts globally and in Vietnam, and collected evidence from previous pandemics such as the SARS-CoV pandemic in 2003. The data were collected from academic journals and reports, verified newspapers, websites of governments and internationally recognised organizations (such as WHO, UNICEF and John Hopkins), which were delivered in the English language. The desk research aimed to: (1) categorise Vietnam’s vulnerable groups who are at risk of poor mental health due to the COVID-19 pandemic, (2) identify COVID-19 stressors on specific groups, and (3) identify the mental health needs of specific groups.

***Stakeholder involvement***

In community-based projects, the definition of stakeholders can be ‘individuals, organisations and community groups who share the same interest in or are affected by the project’s topic. Stakeholders willingly involved in multiple stages of the project to contribute their professional knowledge and/or personal viewpoints towards the research questions and/or the research findings ( Boaz *et al.,* 2018; Patil *et al.,* 2021). We invited different stakeholders to partner with us in 5 phases of the development of the directory.

*Phase 1: Reviewing the desk research findings:* After the desk research, we sought to validate our list of groups most vulnerable to COVID-19 mental health problems by consulting mental health experts and those involved in mental health in Vietnam. The project team invited 5 experts to review the literature review and our categorisation of Vietnam’s vulnerable groups who are severely impacted by the COVID-19 pandemic, with their specific mental health needs.

*Phase 2: Reviewing the list of mental health support services:* The group of experts helped identify the service needs of each group that was identified in Phase 1. Based on those needs, we categorised 3 main types of services to include in the directory, including: (1) Mental health specialised services, (2) COVID-19 related mental health services, and (3) Other health related support. From September 15 to October 22, 2021, we used internet searches and personal contacts to identify organisations with relevant programs and services. This list was then reviewed by the expert partners, plus other non-professional community members with experience of using mental health services. This enabled us to identify gaps and some services which were no longer viable.

*Phase 3: Verifying the first draft of the directory:* The last stakeholder engagement was the verification process. The project team contacted the selected service providers via email, in order to verify information on their programs and services, and add any information that we hadn’t been able to glean from their websites or social media pages. Details we sought from each service provider included: 1. Contact information, 2. Types of service users, 3. Programs and services, 4. Service delivery modes, and 5. Language used in service delivery. To be included within the directory, providers were required to complete a registration form and meet our inclusion criteria which was to be relevant to our service categorisation, and to be able to provide proof of their professional licences or certificates related to mental health.

*Phase 4: Disseminating the directory:* In order to disseminate the directory, we held a series of public talks in Vietnamese and in English. The Vietnamese event was also livestreamed on the OUCRU social media channels. In each event, the guest speakers shared about their mental health struggles and how they overcame them in the context of COVID-19 pandemic. A facilitator supported the questions and answer time and introduced the directory. By hosting these sessions, we aimed to raise awareness about mental health issues, and enable the audiences to share their own experiences. In doing so, they might find support from the community, but also inspire other people who suffer mental health issues. In addition, we found the mental health talk events an effective way to promote the directory and also reach out to service providers that we hadn’t included. Postcards with a QR code linking to the downloadable directory were given out at all the events, and have been distributed in cafes and clinics in Ho Chi Minh city. 

*Phase 5: Updating the directory:* After distribution of the first edition of the directory, some of the service providers who had not responded to us in the verification process (phase 3) heard about the resource and contacted us to update their information. We also received requests from providers that we had not identified before to be included in the directory. We printed and published the 2 ^nd^ version of the mental health directory in April 2022 ( Thai *et al.,* 2022).

***Benefit of stakeholder participation in the project***

In the context of the fourth wave of COVID-19 in Vietnam (since late April 2022), we initiated the project to provide support for Vietnamese communities in response to the arising concern of COVID-19 mental health issues. Due to pressing need for support and the prolonged lockdown in Vietnam, we utilised existing relationships with mental health experts in the validations of the desk research and the list of selected service providers. Collaborating with acquainted stakeholders was beneficial and time efficient for building trust ( Murphy *et al.,* 2021), and our partners were able to respond quickly to our requests to be involved in the project. Having trusting relationships also enabled them to introduce other experts and providers. For example, they helped identify other services that we had not originally considered, including foreign service providers working in the mental health field in Vietnam. Thus, the mental health directory may be a useful resource for not only Vietnamese communities, but also for foreigners living in Vietnam.

Another key value of stakeholder engagement in health research is that it can improve the relevance of research and increase research uptake, by including the different perspectives of stakeholders ( Boaz *et al.,* 2018). In this project, validation from the mental health experts introduced the concept that the mental health of Vietnamese communities has been damaged not only during the COVID-19 time, but prior to the pandemic. Engaging with stakeholders in research promotes transparency, assuring the research findings and the project implementation serves the public interest ( Boaz *et al.,* 2018; Esmail *et al.,* 2015). When seeking the validations by mental health experts the project team aimed to ensure that the data included in the directory were unbiased, multilateral and relevant to the community’s needs. 

Finally, the project team recognized the strength of the stakeholder engagement in helping promote the mental health directory beyond the public events. The guest speakers and participants from the events promoted OUCRU’s mental health directory on social media and across their networks. As a result, more Vietnamese people and mental health service providers have been aware of and been able to access and utilise the directory.

### Reflections on dissemination of the directory

From our perspective, launching the mental health directory through a series of mental health talks was an effective means of approaching the wider community. These public events including well-known personalities and respected community members attracted participants to the events and further assisted to disseminate information about the directory through their own media channels. This recognises the role of media and social media as prominent platforms to access a wider range of stakeholders and audience ( Patil *et al.,* 2021). The face-to-face events also provided an opportunity for the sharing of personal stories of how people in Vietnam struggled and coped with their anxiety, depression and mental stress throughout the prolonged lockdown and the economic recession. Many people spoke about the of lack mental health support and not knowing how to access such support. The directory was presented to them to address this need. The postcards were also effective to direct people to the resource. Twelve months after distribution some cafes still display the postcards and members of the public are accessing the directory using them. 

In conclusion, in response to growing awareness of the mental health impacts from the pandemic ( WHO, 2021), the Public and Community Engagement Department of OUCRU Vietnam partnered with experts and people with lived experience to compile the first directory of mental health services for Vietnamese communities and the expatriate community living in Vietnam. The directory was officially launched in February 2022 and updated in April 2022.

The dissemination of the directory through multiple channels has ensured that it has reached a wider audience – in particular members of the public who may not otherwise have had access to or knowledge of the resource. The directory has been distributed to service providers and people interested in supporting those with mental health needs (including schools, medical facilities, and community groups). The feedback from experts and the wider community has been very positive, confirming that this directory is meeting a current need in Vietnam. We will continue to update and revise the product based on public and service provider feedback, with the aim of supporting Vietnamese communities to access support for their mental health needs.

## Disclaimer

The views expressed in this article are those of the authors. Publication in Wellcome Open Research does not imply endorsement by Wellcome.

## Data Availability

No data are associated with this article. Zenodo: A directory of mental health services for Vietnamese communities and people living In Vietnam. https://zenodo.org/doi/10.5281/zenodo.7870596 ( Thai *et al.,* 2022). Data are available under the terms of the Creative Commons Attribution 4.0 International license (CC-BY 4.0).
