# Supplementary material for: Stakeholder engagement to develop a directory of COVID-19 related mental health services in Vietnam: reflections on a participatory approach
Source: Wellcome Open Res. 2024 Feb 19;9:87. [Version 1] doi: 10.12688/wellcomeopenres.20491.1 (PMC11292178; doi:10.12688/wellcomeopenres.20491.1)
Supplement: Peer review history

# Reviewer report: Reviewer response for version 1

**DOI:** 10.21956/wellcomeopenres.22684.r83770

**Published:** 2024-07-31

**Authors:** Marciana Popescu

**Affiliations:** 1 Fordham University, New York, New York, USA

This article presents a very welcome initiative contributing to increased access to existing mental health services for the Vietnamese communities, as it was premised that these communities faced multiple mental health challenges rooted in direct pandemic experiences, and indirect racial discrimination, that surfaced during and post pandemic. Focusing on the literature that points to a need for participatory approaches, and engagement with multiple stakeholders, the project presented in this article however proceeded with a very narrow definition of stakeholders. It is not clear to what extent - if at all - were the actual communities included, as primary stakeholders. While it is important to create a directory of resources - it is equally important to engage the voices of the people that are potentially using these resources - and this part is not sufficiently described. 

 Either way, it would be useful to list all the stakeholders engaged in the project - and maybe the selection/inclusion criteria that were used. Based on the information included here, it seems that the project included primarily mental health experts and service providers - while engaging with media to raise awareness on the issue and services available. There is some reference to non-professional community members reviewing the list of mental health services available; and one line in the Reflections on Dissemination section mentioning a partnership with people with lived experience - I think the readers will benefit for a more elaborate description of such stakeholders and the extent in which they were included throughout the project.

 As a next step, it might be useful to assess the extent to which the directory has led to an increase in access and utilization of existing services, including the perspectives of Vietnamese communities for which the directory was created.

 The project is a great initiative, as information is needed - but could be expanded, to highlight participatory, inclusive methods that were used, and ways in which they will continue to inform research and practice.

Does the article adequately reference differing views and opinions?

Partly

Are all factual statements correct, and are statements and arguments made adequately supported by citations?

Yes

Is the Open Letter written in accessible language?

Yes

Where applicable, are recommendations and next steps explained clearly for others to follow?

Yes

Is the rationale for the Open Letter provided in sufficient detail?

Yes

Reviewer Expertise:

Migration policy, forced migration, women's rights, evaluation research.

I confirm that I have read this submission and believe that I have an appropriate level of expertise to confirm that it is of an acceptable scientific standard.

# Reviewer report: Reviewer response for version 1

**DOI:** 10.21956/wellcomeopenres.22684.r83768

**Published:** 2024-06-11

**Authors:** Sam Miles

**Affiliations:** 1 Queen Mary University of London, London, England, UK

This is a timely and very interesting research article that discusses how stakeholder engagement in Vietnam helped to develop a directory of Covid-19 based mental health services.

 This article focuses on a strongly participatory approach to develop better signposting to mental health resources for Vietnamese community members seeking support. A strength here is the engagement of community members and others with lived experience, and reflection on that process.

 You also included an interesting sub-focus on Vietnamese diaspora communities, including Vietnamese students studying abroad, and the burden associated with diaspora populations being seen as somehow culpable for the pandemic because of its Asian origin.

 Your precis of stakeholder engagement suggests it was a comprehensive and meaningful exercise, with collaborators engaged in every stage of the research.

 The incorporation of postcards with a QR code for people to link to a downloadable mental health support services directory is innovative; I wonder if you have some data you could add on numbers of click-throughs and downloads, and split data by different geographical zones in Ho Chi Minh City to ascertain where need is greatest (or, conversely, where mobile technology/mobile data capacity is most dominant).

 Relatedly, you rightly recognise the huge value of media and social media as platforms to engage with larger and more diverse audiences than may have been possible before, and that the engagement of different community members, distributing the directory resource through their own social media networks, will be useful in widening the range and access of the resource.

 This is valuable, important research and anything that strengthens take-up of mental health services in a range of settings - and from diverse stakeholders/clients - is to be applauded.

 Small issues:

 Grammar mistake in Introduction section, ‘Continuing it’s’.

 Missing a closing quotation mark in ‘Stakeholder involvement’

Does the article adequately reference differing views and opinions?

Yes

Are all factual statements correct, and are statements and arguments made adequately supported by citations?

Yes

Is the Open Letter written in accessible language?

Yes

Where applicable, are recommendations and next steps explained clearly for others to follow?

Yes

Is the rationale for the Open Letter provided in sufficient detail?

Yes

Reviewer Expertise:

Participatory approaches; stakeholder engagement; PPI and co-production; COVID-19; migrant workers; vulnerable communities; medical sociology

I confirm that I have read this submission and believe that I have an appropriate level of expertise to confirm that it is of an acceptable scientific standard.
